# Notes and News

**Published:** 1906-01-06

**Authors:** 


### Notes and News.

The Princess Henry of Battenberg will open on Friday the Babies' Ward at the Belgrave Hospital for Children, Kennington. The ward contains eight cots, and has hitherto remained unoccupied, owing to want of funds; but the Ladies' Association, of which Princess Henry is patron, have undertaken to provide sufficient money to defray the cost for one year.

Lady Minto, wife of the Viceroy of India, has taken over the duties of Lady President of the National Association for Providing Female Medical Aid to the Women of India.

Mrs. Ada Lewis-Hill has provided for the year 1905 her annual gift of £10,000 to King Edward's Hospital Fund, which has also received the League of Mercy's contribution for the year 1905, amounting to £15,000.

The terms of the Bawden Trust Fund have now been completed, and the securities, representing the £100,000 given by Mr. E. G. Bawden for the benefit of the charities mentioned in Mr. Edgar Speyer's letter, published in our issue of September 9 last, have been transferred to Mr. Edgar Speyer, Lord Hillingdon, and Lord Revelstoke as trustees.

We are informed that an association of holders of the assistants' certificate of the Society of Apothecaries of London is being organised. The object of the association is to enable the certificate holders, by combining, to protect the special privileges which belong to them. For this purpose an appeal is being made to all who are interested in the matter, and especially to those holding appointments in the public and hospital services. The support of the Society of Apothecaries has been already promised. There are good grounds for hoping that the Master and Wardens will place a room at the Society's hall at the disposal of the proposed association where meetings may be held for the purpose of discussing the several questions which are said to be agitating the whole dispensing body. All who are willing to join the association should communicate with Mr. Albert Howell, Hackney Union Dispensary, Rosebery Place, Dalston, N.E.

Lord Rothschild has accepted the office of President of the Royal Dental Hospital of London, and Sir Frederick Treves has consented to become consulting surgeon to the same institution.

The New Year Honours include the names of Surgeon-General W. R. Browne, M.D., Indian Medical Service, Surgeon-General with the Government of Madras, who becomes a Companion of the Order of the Indian Empire; and Lieut.-Colonel Robert Shore, M.D., Indian Medical Service, Residency Surgeon, Mewar, who receives the Kaisar-i-Hind medal.

A Blue-book has been issued containing a statistical report of the health of the Navy for the year 1904, and the information it contains must be regarded as satisfactory in the highest degree. The invaliding ratio of the total force was 22.7 per thousand, and shows a decrease of 7.28 as compared with the average for the last seven years. The death-rate was 4.45 per thousand, as compared with an average of 5.46 per thousand for the last seven years. The highest invaliding and death-rates were on the East Indies station.

At Marylebone County Court on Monday a case of some moment to men who shave, or are shaved, was heard before Judge Selfe and a special jury. The plaintiff, a male nurse, claimed damages from the proprietor of a hairdresser's shop in West London on the ground that whilst he was being shaved he contracted a skin complaint. The defence was that the plaintiff did not suffer from the complaint, and on his behalf an assistant was called, who said that sometimes carbolic acid was used as an antiseptic. He added that he did not use it, "because it was not obligatory," but only used hot water and soap. Another witness for the defence was a member of the House of Commons, who stated that he had often been shaved at the shop in question, and intended to continue the practice, a remark which prompted the plaintiff's counsel to ironically wish him "A happy New Year." A second barber having mentioned that "he had separate mugs for his less cleanly customers," and "that some fussy people kept their own mugs," the Judge submitted the case to the jury to decide, mentioning that it was one of peculiar interest to him, because he himself was in the habit of visiting barbers' shops. The jury found for the plaintiff, and awarded him £15 damages. Possibly the plaintiff's success will help to teach a much-needed lesson to a large class of persons who have it in their power, by carelessness or wilful neglect, to propagate infectious and loathsome maladies. The safest thing is for a man to shave himself, but if he pays someone else to do it for him he ought not to be exposed to the risk of contracting skin disease. It is easy and inexpensive to keep razors, brushes, and mugs in a clean state; and no one who cannot do this is fit to pursue the calling of a barber. We are afraid, however, that the inspection by experts of the conditions under which the average knight of the razor carries on his trade would result in revelations sufficient to frighten away most of the customers.

